# Different challenges, different approaches and related expenditures of community-based tuberculosis activities by international non-governmental organizations in Myanmar

**DOI:** 10.1186/s40249-017-0263-9

**Published:** 2017-03-24

**Authors:** Wai Wai Han, Saw Saw, Petros Isaakidis, Mohammed Khogali, Anthony Reid, Nguyen Hoa, Ko Ko Zaw, Si Thu Aung

**Affiliations:** 1 Department of Medical Research, Ministry of Health and Sports, No 5, Ziwaka Road, Dagon Township, 11191 Yangon, Myanmar; 2 Médecins sans Frontières, Operational Research Unit, Luxembourg city, Luxembourg; 3 Centre for Operational Research, International Union Against Tuberculosis and Lung Disease, Paris, France; 4 National Tuberculosis Programme, Ministry of Health and Sports, Nay Pyi Taw, Myanmar; 5 National Tuberculosis Programme, Hanoi, Vietnam

**Keywords:** Operational research, Cost, Sustainability, Budget allocation

## Abstract

**Background:**

International non-governmental organizations (INGOs) have been implementing community-based tuberculosis (TB) care (CBTBC) in Myanmar since 2011. Although the National TB Programme (NTP) ultimately plans to take over CBTBC, there have been no evaluations of the models of care or of the costs of providing CBTBC in Myanmar by INGOs.

**Methods:**

This was a descriptive study using routinely-collected programmatic and financial data from four INGOs during 2013 and 2014, adjusted for inflation. Data analysis was performed from the provider perspective. Costs for sputum examination were not included as it was provided free of charge by NTP. We calculated the average cost per year of each programme and cost per patient completing treatment.

**Results:**

Four INGOs assisted the NTP by providing CBTBC in areas where access to TB services was challenging. Each INGO faced different issues in their contexts and responded with a diversity of strategies. The total costs ranged from US$ 140 754 to US$ 550 221 during the study period. The cost per patient completing treatment ranged from US$ 215 to US$ 1 076 for new cases and US$ 354 to US$ 1 215 for retreatment cases, depending on the targeted area and the package of services offered. One INGO appeared less costly, more sustainable and patient oriented than others.

**Conclusions:**

This study revealed a wide variety of models of care and associated costs for implementing CBTBC in diverse and challenging populations and contexts in Myanmar. Consequently, we recommend a more comprehensive evaluation, including development of a cost model, to estimate the costs of scaling up CBTBC country-wide, and cost-effectiveness studies, to best inform the NTP as it prepares to takeover CBTBC activities from INGOs. While awaiting evidence from these studies, model of CBTBC that have higher sustainability potential and allocate more resources to patient-centered care should be given priority support.

**Electronic supplementary material:**

The online version of this article (doi:10.1186/s40249-017-0263-9) contains supplementary material, which is available to authorized users.

## Multilingual abstract

Please see Additional file 1 for translations of the abstract into the six official working languages of the United Nations.

## Background

Although the global mortality rate of tuberculosis (TB) in 2015 was 47% lower than in 1990, TB now ranks alongside the human immune deficiency virus (HIV) as a leading cause of death worldwide [1]. In 2014, an estimated 9.6 million people developed TB and 1.5 million died from the disease (including 0.4 million who were HIV co-infected) [1]. Myanmar is classified by the World Health Organization (WHO) as one of the 30 TB, TB/HIV and MDR-TB high-burden countries with a TB incidence of 373/100 000 population [2]. Consequently, TB control has been one of the priorities in Myanmar’s national health plan.

Additionally, some population groups are of particular concern as they may be contributing to the ongoing infection rate. These include Internally Displaced Persons (IDPs), urban slum dwellers and hard- to-reach populations, especially those living in rural areas, hilly regions and border areas [3]. These populations maintain reservoirs of infection and continue to spread the disease in the community. Therefore, in 2011 the NTP in collaboration with international non-governmental organizations (INGOs), started to implement a community-based TB care (CBTBC) programme for active case finding (ACF) in two regions and three states [3]. The aim of ACF is to identify TB infected patients, to initiate treatment and ensure follow-up until completion [3].

The groups of concern range widely in their needs and the strategies required to perform active case finding. Urban slum dwellers are easier to reach physically but may be difficult to locate in the chaos of the slums. People living in remote mountainous areas pose a physical challenge in locating and maintaining contact over time. Thus, although active case finding is the goal, how to achieve this in some contexts can be challenging. The four INGOs in Myanmar each approach their target population using different strategies, adapted to the population’s unique circumstances. As the NTP ultimately hopes to take over CBTBC, it is important to document the strategies employed and the costs associated with delivering the care. To date there have been no evaluations of the care nor of the costs of providing ACF in Myanmar by INGOs, although recent studies in Cambodia pointed out that community-based active case finding and ACF targeting household and neighborhood contacts are highly cost-effective, with the additional benefit of early case finding of patients from vulnerable age groups, i.e., younger and older [4, 5].

Thus, the aim of this study is to describe the differences in provision of CBTBC and associated costs by four INGOS in Myanmar over the period of 2013 and 2014.

## Methods

### Design

This is a descriptive study using routinely-collected programmatic and financial data from four INGOs.

### General setting

Myanmar is a multi-ethnic country located in South East Asia with the population of 51 million. It is bordered by India, Bangladesh, China, Laos and Thailand. There are over 100 languages and dialects spoken in Myanmar, which contribute to language barriers for health service delivery. In addition, the geography of the country, with a number of rivers and mountains, make many areas of the country hard to access [6], creating further barriers to delivering health care. The country is administratively divided into the Nay Pyi Taw Council Territory and 14 States and Regions. Administratively, the States and Regions are the same, but geographically, the States are situated along the border areas and have more ethnic populations while the Regions are situated in the center of the country. Generally, the Regions are more urbanized than the States. Seventy five percent of the population live in rural areas and only 25% are urban inhabitants [7].

The Department of Public Health and Department of Medical Services are the service providers and regulators in protecting the health of the people [8]. However, INGOs have been playing an increasingly important role within the evolving political and administrative context. The government used to be the main source of financing, with provision of services virtually free until user charges were introduced in the form of cost sharing in 1993; since then, household out-of-pocket payment has become the main source of finance of the health system. Health care is now highly fragmented both in provision and financing [8].

### Specific setting

The NTP is the main provider of TB control and care services. It runs TB centers in 15 Regions and States, with 101 TB teams at district and township level. However, there are no TB centers or teams at the ward, village tract and village level [2].

### Community based TB care

A number of implementing partners, both local and international NGOs, are currently implementing CBTBC on a large scale across the country, with the financial support from international donors. The core elements of CBTBC being implemented by the partners are the same. Given different contexts and circumstances, however, their approaches vary widely. The elements of CBTBC include: 1) community mobilization, 2) recruitment of community volunteers, 3) training of community volunteers, 4) TB awareness-raising within communities through volunteers, 5) detection of TB suspects by volunteers, 6) attending DOT by the volunteers until finishing the course of TB treatment, 7) counseling TB patients for treatment adherence, and 8) support for TB patients (transportation, nutritional support, incentives) [3].

### Sources of data, data variables and data collection

All four INGOs provided TB programme monitoring and evaluation reports, activity reports and financial reports that became the data sources. Programme costs included human resources, training, communication materials and community awareness-raising, health products and equipment, TB patient supports, monitoring and evaluation activities, planning and administration and overhead. The components of human resource costs included salaries for TB staff of each INGO, both local and international, and incentives or payment for the community volunteers. Training costs were comprised of fees for trainers, food, travel and accommodation costs for the trainers and trainees, costs for learning aids and stationery, and fees for renting training venues. Communication materials involved expenses for production and delivery of IEC materials, costs for community awareness-raising activities and social mobilization. The costs for health products and medical equipment included procurement of medicines to treat side effects and multivitamins and establishment and maintenance of microscopic laboratories. Costs incorporated into TB patient supports were transportation and meal allowances for patients and volunteers during outpatient visits. Other costs included chest X-rays, supplementary food support, plus transportation and meal allowances for the patients and attendants during inpatient hospital stays. Costs for monitoring and evaluation included travel and accommodation for TB staff of INGOs and NTP officials for supervision visits, as well as costs for monthly volunteer meetings, quarterly experience sharing meetings, mid-term and annual evaluation meetings. Costs for planning and administration included the expenses for office supplies, stationery and printing costs. Overhead costs included cost for office rental, vehicle rental, fuel and maintenance costs, and telecommunications. The cost for anti-TB medicines were obtained from NTP’s programme report [9].

In this study, we did not include cost for sputum examination as the procedure is identical in all models of care.

### Analysis

A descriptive analysis was performed adopting a health service provider perspective. A data extraction sheet was prepared to collect the relevant data from the reports of INGOs. We calculated average cost per year of each INGO and cost per patient completing treatment. As this study includes costs for the years 2013 and 2014, cost data from 2013 were adjusted for inflation and expressed in 2014 constant prices. For staff and community volunteers jointly using other services, their salaries or incentives were allocated proportionally, based on the time spent for community based TB care activities.

## Results

**Fig. 1:**
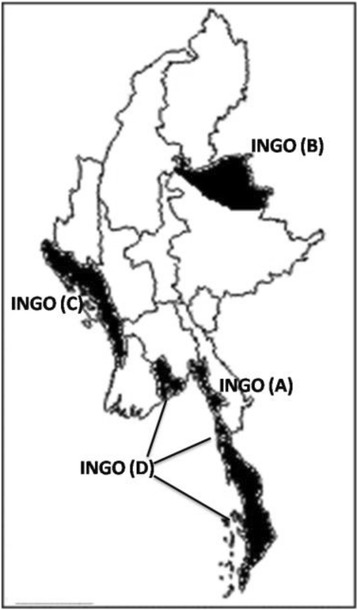
States and Regions of Myanmar where four INGOs are implementing community based TB care (2013-14)

Four INGOs provided community based TB care in 22 townships in Myanmar during the study period (Fig. 1). The respective models of care, populations served and types of services of the INGOs are shown in Table 1.

**Table 1:** Models of care, populations served and types of services of four international NGOs involved in community based TB care in Myanmar, 2013-14

INGOs	(A)	(B)	(C)	(D)
Model of health care delivery	Newly recruited community health volunteers (TB + HIV)	Existing community health volunteers (CHV) (TB+ malaria)	Newly recruited community health volunteers with diagnostic facilities	Established self help groups (SHG) with TB patients, family of TB patients and other community volunteers
Model description	From village-based mobility working groups, outreach workers were identified, recruited and trained for CBTBC	Existing CHVs were used for delivery of CBTBC	• Established TB screening clinics and mobile teams to detect and diagnose TB. • DOT providers were recruited and trained to detect TB suspects and follow patients	Self Help Groups were formed and trained with the intention of delivering CBTBC with their own fund through livelihood activities
Target population	Migrants and mobile populations	Rural heard-to-reach population	Rural population including IDP^a^	Urban slums
Population size	1 434 500	726 500	869 750	1 432 470
Volunteer payment	Salary	Travel, meals, and accommodation and costs to accompany patients for diagnostic and follow up visits	Performance- based payment based on number of TB patients referred and treated	SHGs got benefit from their livelihood activities
Average number of volunteers per year	117	796	436	157
Detection of TB suspects and referral	• Health education in community and migrant settings to detect TB suspects and refer them to township health departments for diagnosis. • Active TB case finding by mobile team • Contact tracing among household members	• CHVs refer people with TB symptoms to township health departments for diagnosis. • Contact tracing among household members	• DOT providers detect TB suspects in their community and refer them to INGO’s primary health care clinic and TB clinics for diagnosis • Diagnosed TB cases referred to township health department for TB treatment. • Contact tracing among household members	• Health education in community and migrant’s worksites to detect TB suspects and refer them to township health departments for diagnosis. • Contact tracing among household members
Provision of DOTS	• Outreach Health Workers (OHWs)deliver anti-TB medicines to patients monthly • OHWs provide DOTS during the first week of treatment	• Outreach Health Workers (OHWs)supply anti-TB medicines to patients monthly • OHWs provide DOTS during the first week of treatment	• DOT providers supply anti-TB medicines to patients monthly • DOTS provided to most patients until the end of treatment	• Self-help groups supply anti-TB medicines to patients monthly DOT provided during the first week of treatment. • DOT provided until the end of treatment in selected areas
Treatment monitoring	Monthly	Monthly	Monthly	Monthly
Patient support	• Food • Transport fees • Costs for investigations • Meal allowance during outpatient and inpatient visits • Side effect medications	• Food • Transport fees • Costs for investigations • Meal allowance during outpatient and inpatient visits	• Food • Transport fees • Costs for investigations • Meal allowance during outpatient and inpatients visit • Side effect medications	• Food • Transport fees • Costs for investigations • Meal allowance during outpatient and inpatient visits • Side effect medications
Health education	• Individual discussion with patients • Group health talks • Health education through FM radio	• Individual discussion with patients	• Individual discussion with patients • Group health talks	• Individual discussion with patients • Group health talks • Behavior change communication activities within community

^a^ *IDP* internally displaced populations

Table 2 shows the average cost per year and cost for different programmatic areas of community-based TB care by four INGOs for the years 2013 and 2014. The average cost per year ranged from US$ 140 753 to US$ 550 221. INGOs (A) and (C) spent a large share of their budget for staff salaries (45% and 60% respectively). INGO (B) spent mostly on trainings and INGO (D) spent the largest proportion of its budget for patient support. The proportion of the budget allocated for monitoring and evaluation was similar among all INGOs.

**Table 2:** Average cost per year and itemized costs for community-based TB care by four INGOs in Myanmar, 2013^a^ -2014

Cost items	INGOs			
	(A) Cost in US$ (%)	(B)	(C)	(D)
Total cost	550 221	140 754	316 173	239 650
Human resource				
Staff salary	248 555 (45)	26 568(19)	190 099 (60)	59 241(25)
Volunteer incentive	40 142 (7)	8 878 (6)	4 372 (1)	19 999(8)
Training				
Volunteers	14 438 (3)	27 596 (20)	2 001 (1)	9 945 (4)
INGO staff		3 946 (5)	2 266 (1)	8 989 (4)
Communication materials	15 236 (3)	5 338 (4)	4 111 (1)	7 059 (3)
Health products and equipment	12 514 (2)	0	24 090 (8)	427(0.2)
Patient support	92 820(17)	29 238 (21)	26 007 (8)	99 585 (42)
Monitoring and evaluation	25 194 (5)	3 649 (3)	15 368 (5)	11 937 (5)
Planning and administration	31 463 (6)	0	4 888 (2)	14 275 (6)
Overhead and general operational expenses	69 859(13)	32 148 (23)	42 971 (14)	8 193 (3)

^a^cost data from 2013 were adjusted for inflation and expressed in 2014 constant prices

The number of TB cases detected, the number of patients completing treatment and the cost per patient completing treatment through community based TB care are shown in Table 3. Drug costs were incurred by National TB Programme and it cost US$ 22.5 per new case and US$ 161 per relapse case to complete treatment [9]. The average cost per case that completed treatment ranged from US$ 215 to US$ 1 076 for new cases and US$ 354 to US$ 1 215 per relapse cases.

**Table 3:** Average number of TB cases detected, number of patients completing treatment and cost per patient completing treatment through community-based TB care by four INGOs in Myanmar, 2013^a^ and 2014

Variables	INGO			
	(A)	(B)	(C)	(D)
Number of TB cases detected per year	2 605	206	514	1 490
Number of TB patients that completed treatment per year	1 936	134	371	1 242
Cost per patient that completed treatment (new case)^a^	306	1 076	874	215
Cost per patient that completed treatment (retreatment)^a^	445	1 215	1 013	354

^a^Cost in US$. cost data from 2013 were adjusted for inflation and expressed in 2014 constant prices

## Discussion

To our knowledge, this is the first study describing different approaches to community-based TB care (CBTBC) and associated costs by international NGOs in Myanmar. In the analysis we considered all the costs incurred by INGOs to deliver community based TB care during 2013 and 2014. This does not include the cost of sputum examination as it was provided free of charge by NTP to the patients and the procedures were identical in all models of care. All four INGOs were assisting the NTP by implementing CBTBC in areas where access to TB services was challenging. The study revealed significant diversity in approaches and strategies among the different INGOs and a relatively wide range of average cost per year, from US$ 140 754 to US$ 550 221 overall during the study period. In the view of the expected eventual handover of these activities to NTP, the findings presented here may inform the development of a model to estimate the cost of scaling up CBTBC country-wide and cost-effectiveness studies and contribute to the future planning and budgeting exercises of the NTP.

INGO (A) delivered services in the areas where there are large migrant populations. It incorporated mobile team activities in its active case detection in addition to a volunteer-based programme [10]. It covered a relatively larger population. These factors could explain why INGO (A) had a relatively high number of cases detected and, obviously, high total costs. However, in terms of cost per patient completing treatment, INGO (A) had relatively lower costs.

INGO (B) implemented CBTBC in the hilly regions with a sparse population and difficult transportation. Active case detection was performed by training existing local community health workers. Although the total cost of operating CBTBC by INGO (B) was found to be relatively lower than the others, the cost per patient completing treatment was the highest [more than five times that of INGO (D)]. This may be due to the context in which INGO (B) operates.

INGO (C) worked in one of the conflict-affected areas in Myanmar and therefore it mainly provided care to internally displaced persons. Similar to INGO (A) it carried out active case detection via mobile teams and community volunteers. On top of that, it established its own diagnostic facilities. It had the second highest total cost and cost per patient treated, again probably due to the context in which it operated.

INGO (D) provided care in settings similar to INGO (A) and the population served was also comparable. In contrast to other INGOs, it delivered community-based TB care by establishing self-help groups (see Table 1). It was able to detect a large number of cases and achieved the lowest cost per patient treated among the four INGOs.

As there are no standard tools to assess the performance of CBTBC in Myanmar, we were unable to evaluate the overall performance of each INGO based on standard indicators. Neither were we able to assess individual elements of the interventions that were carried out. Instead, we used existing data from qualitative and mixed-methods studies that have been conducted in Myanmar to appraise the work of the INGOs in terms of their potential sustainability [11, 12].

Three of four INGOs, (A, B and D) supported the NTP within its definition of the “role and responsibilities of implementers in delivering CBTBC” (4). However, INGO (C) operated in parallel to NTP by providing CBTBC with their own diagnostic facilities. The number of presumptive TB cases detected and the number of TB patients completing treatment were relatively higher in INGOs (A) and (D) while INGO (B) reported the highest costs per patient treated, which may be explained by the fact that it targeted communities in some of the most hard-to-reach areas of the country.

The study revealed several interesting findings regarding sustainability, mainly related to INGO infrastructure and the involvement of community members. First, infrastructure created by external partners, such as the diagnostic facility run by INGO (C), would be hard to sustain without funding support from the INGO. Handing over such facilities to NTP would result in unnecessary duplication, given that the NTP already has its own network of facilities in these areas.

Second, as sustainability is an important issue in CBTBC, some INGOs performed various measures to ensure that the delivery of CBTBC could be continued after their withdrawal. INGO (B) trained and utilized existing local community health workers to continue delivery of CBTBC after their withdrawal. However, the community health workers were also required to perform other tasks such as environmental sanitation, health education, nutrition surveillance, malaria case finding and treatment, case management of pneumonia and diarrhoea in children under five, and identification of pregnancy at risk for referral according to community’s need [6]. This workload may have diluted the effectiveness of CBTBC by that INGO. On the other hand, using existing community health workers could result in economies of scale. However, INGO (B) spent more than other INGOs for a TB patient to be successfully treated. INGO (A) and (D) formed local groups to help TB patients in preparation of their future withdrawal from the country. INGO (A) formed village mobility working groups (VMWGs), which were expected to recruit and support volunteers who can provide CBTBC in their respective areas with the groups’ own funds. Similarly, INGO (D) formed self-help groups (SHGs) to look after TB patients and take over CBTBC when the supporting INGO phased out. Consequently, these SHGs performed fund raising activities alongside CBTBC. A study assessing the cost of implementing SHGs showed that half of them were able to mobilize funding to cover up to 40% of the costs for TB patient support [13]. Furthermore, a previous study has shown that the majority of volunteers of INGO (D) stayed with the organization longer than those of INGO (A) (four vs. three years) [12]. The study on effectiveness of SHGs showed that 92% of the members remained in SHGs after completion of TB treatment [11]. These findings suggest that the CBTBC model used by INGO (D) may be more sustainable.

A study conducted in Zambia and Rwanda, which assessed the cost of community-based programmes for HIV control, indicated that the highest cost was for direct project services such as health education, referrals, primary health care services and provision of nutrition (7). In our study, only INGO (D) allocated more of their budget to patient support whereas INGOs (A) and (C) spent mostly on staff salaries.

Total costs were found to be the highest for INGO (A) and the lowest for INGO (B). However, the cost per patient completing treatment was found to be relatively higher for INGOs (B) and (C) than INGOs (A) and (D). Based on the study assessing involvement of community volunteers in TB control, the socio-demographic characteristics of the target population and population size of coverage areas of INGO (A) and INGO (D) are quite similar [12]. But, INGO (D) spent less than INGO (A) per patient to complete treatment. Thus, based on the available evidence, INGO (D) appeared to show the allocation of their financial resources more patient-oriented.

Our study has several strengths and limitations. First, this was a comprehensive study that mapped and described all INGOs providing community-based TB care in Myanmar. Second, we were able to obtain financial data from all INGOs using a standardized data collection tool, which allowed for broad cost comparisons. A limitation of this study was that we were unable to ascertain or validate the cost data obtained from the INGOs. However, as the INGOs in the country have their own internal auditing mechanisms, we are confident that the integrity and validity of the data used in this study is acceptable. There are a number of international studies evaluating the cost and cost effectiveness of community- based TB care, however, the ability to compare our data to international literature is limited due to the differences in models of care and contexts. Studies across Africa found the cost per patient of completing treatment under community -based TB care were quite varied, from US$ 60.7 in Ethiopia, US$ 128 in Tanzania and US$ 726 for new cases and US$ 1 419 for retreatment cases in South Africa [14–16]. Studies done in Bangladesh and Brazil showed that it cost US$ 64 and US$ 548 respectively for a patient to complete treatment by community-based TB care [17, 18]. The cost for community-based TB care in Myanmar seemed relatively higher than most of the previous international studies. However, these other studies were conducted among populations residing in urban and densely populated rural area which might reduce the transportation and other management costs. Again, the overhead and general operational expenses of INGOs included in our study made the CBTBC more expensive.

## Conclusion

In conclusion, the findings from this study may inform the NTP about the models of community-based TB care and their associated costs. We recommend that standardized tools to evaluate the CBTBC performance should be developed and comprehensive evaluations, including the development of a model to estimate the cost of scaling up CBTBC country-wide and cost-effectiveness studies, should be carried out, especially as CBTBC activities are eventually to be taken over by the government. We suggest that while awaiting evidence from cost-effectiveness studies, models of CBTBC that have a higher sustainability potential and that allocate the largest share of their resources to patient-centered care should be supported.

## Additional file

Additional file 1: Multilingual abstract in the six official working languages of the United Nations. (PDF 643 kb)

## Abbreviations

ACF: Active case finding
CBTBC: Community –based tuberculosis care
DOT: Direct observation treatment
DOTS: Directly observed treatment short course
FM: Frequency modulation
HIV: Human immunodeficiency virus
IDP: Internally displaced persons
IEC: Information Education and Communication
INGOs: International Nongovernmental Organizations
MDR-TB: Multidrug resistant tuberculosis
NTP: National tuberculosis program
SHGs: Self help groups
TB: Tuberculosis
VMWGs: Village mobility working groups
WHO: World Health Organization
