# Supplementary material for: Different challenges, different approaches and related expenditures of community-based tuberculosis activities by international non-governmental organizations in Myanmar
Source: Infect Dis Poverty. 2017 Mar 24;6:59. doi: 10.1186/s40249-017-0263-9 (PMC5364660; doi:10.1186/s40249-017-0263-9)
Supplement: Additional file 1: — Multilingual abstract in the six official working languages of the United Nations. (PDF 643 kb) [file 40249_2017_263_MOESM1_ESM.pdf]

Translation of the abstract into the six official working languages of the United Nations

التحديات المختلفة و النهج المختلف والنفقات المرتبطة بالأنشطة المجتمعية لمرض السل المقدمة من قبل المنظمات الدولية غير الحكومية في ميانمار

واي واي ، ساو ساو ، بيتروس ايساكيديس ، محمد خوجلي ، انطوني ريد ، نين هاوا ، كو كو زاو ، سي ثو انغ.

ملخص الدراسة
خلفية الموضوع: تقوم المنظمات الدولية غير الحكومية (INGOs) بتقديم الرعاية المجتمعية لداء السل (TB) منذ عام 2011. وعلى الرغم من أن البرنامج الوطني لمرض السل (NTP) يخطط لأن يحل محل الرعاية المجتمعية CBTBC في نهاية المطاف، لم يكن هناك أي تقييمات لنماذج الرعاية وتكاليف تقديم الرعاية المجتمعية لمرض السل في مينمار من قبل المنظمات الدولية الغير حكومية INGOs.
منهجية البحث: كانت هذه دراسة وصفية باستخدام البيانات البرنامجية والمالية التي تم جمعها بشكل روتيني من أربع منظمات دولية غير حكومية INGOs خلال العام 2013 والعام 2014 وهي معدلة بسبب التضخم. تم إجراء تحليل البيانات من وجهة نظر مقدم الخدمة. لم يتم ادراج تكاليف فحص البلغم إذ أنها تقدم مجاناً من قبل البرنامج الوطني لمرض السل NTP. حسبنا متوسط التكلفة السنوية لكل برنامج والتكلفة لكل مريض يكمل علاجه.
النتائج: ساعدت أربع منظمات دولية غير حكومية البرنامج الوطني لمرض السل من خلال تقديم الرعاية المجتمعية لمرض السل CBTBC في المناطق التي يصعب فيها الحصول على علاج لمرض السل. تواجه كل منظمة غير حكومية قضايا مختلفة وكانت الإستجابة لهذه القضايا من خلال تقديم مجموعة متنوعة من الاستراتيجيات. وتراوحت التكاليف الإجمالية من الولايات المتحدة الأمريكية ما بين 140.754 دولار إلى 550.221 دولار خلال فترةهذه الدراسة. وتراوحت تكلفة علاج كل مريض لايزال يكمل العلاج مابين 215 دولار إلى 1.067 دولار للحالات الجديدة و 354 دولار إلى 1215 دولار لحالات إعادة العلاج حسب المنطقة المستهدفة ومجموعة الخدمات التي تم تقديمها. تبين أن هناك واحدة من هذه المنظمات الدولية الغير حكومية المشاركة في الدراسة تقدم برنامجاً أقل تكلفة وأكثر استدامة وتتمحور أكثر من غيرها حول المريض.
الخاتمة: كشفت هذه الدراسة مجموعة واسعة من نماذج الرعاية والتكاليف المرتبطة بها لتنفيذ خطة تقديم الرعاية المجتمعية لمرض السل CBTBC في سياقات ومناطق متنوعة وصعبة في مينمار. ونتيجة لذلك فإننا نوصي بتقييم أكثر شمولاً يتضمن تطوير نموذج التكلفة لتقدير تكاليف رفع مستوى تقديم الرعاية المجتمعية لمرض السل CBTBC في عموم البلاد ودراسات فعالية التكلفة لكي يستفيد البرنامج الوطني لمرض السل عندما يحل محل الأنشطة المقدمة من قبل المنظمات الدولية الغير حكومية التي تقدم الرعاية المجتمعية لمرض السل. وفي انتظار الدليل من هذه الدراسات يجب أن تعطى أولوية الدعم للنموذج الذي قدم رعاية مجتمعية لمرض السل أكثر استدامة وخصص موارد أكثر للرعاية التي كان محورها هو المريض.

Translated from English version into Arabic by Randa82, through

TRANSLATORS
WITHOUT BORDERS

国际非政府组织在缅甸开展以社区为基础的结核病活动面临的不同挑战、使用的不同方法及相关支出

Wai Wai Han, Saw Saw, Petros Isaakidis, Mohammed Khogali, Anthony Reid, Nguyen Hoa, Ko Ko Zaw, Si Thu Aung

摘要
引言：国际非政府组织（INGOs）自 2011 年起在缅甸实施基于社区的结核病护理（CBTBC）。尽管国家结核病项目（NTP）最终计划接管 CBTBC，但尚未对 INGOs 在缅甸提供 CBTBC 护理模式或花费进行过评估。
方法：我们对 4 个 INGOs 2013、2014 年定期收集项目数据和财务数据进行描述性研究，相关数据根据通货膨胀进行调整。从提供者的角度进行数据分析。因痰液检查费用由 NTP 免费提供所以不包括在内。我们计算了每个项目的平均成本和每个患者完成治疗的成本。
结果：4 个 INGOs 协助 NTP 在难以获得结核病服务的地区提供 CBTBC 援助。各 INGO 在不同环境中面临不同的问题，并采取相应的解决策略。在研究期间，总成本为 140 754-550 221 美元。根据目标地区和提供的一揽子服务，每例患者完成治疗的成本为，新病例从 215 美元到 1 076 美元，复治病例从 354 美元至 1 215 美元不等。其中一个 INGO 相比其他 3 个，具有成本较低、更可持续和更面向患者的优势。
结论：本研究揭示了在缅甸各种具有挑战性的人群和背景下，实施多种 CBTBC 护理模式及其相关成本。因此，我们建议进行更为全面的评估，包括成本估算模型的开发，扩大 CBTBC 全国范围的成本估算和成本效益研究，以便在 NTP 准备从 INGO 接管 CBTBC 时，为 NTP 提供最佳信息。在等待这些研究证据时，应优先支持 CBTBC 模型中具有较高可持续发展潜力和以患者为中心分配更多资源的护理模型。

Translated from English version into Chinese by Yin-Long Li, edited by Yang Pin, through

CHINESE CENTER FOR DISEASE CONTROL AND PREVENTION
NATIONAL INSTITUTE OF PARASITIC DISEASES

Les différentes difficultés et approches, ainsi que les dépenses connexes des activités communautaires d'organisations internationales non gouvernementales visant le traitement de la tuberculose au Myanmar

Wai Wai Han, Saw Saw, Petros Isaakidis, Mohammed Khogali, Anthony Reid, Nguyen Hoa, Ko Ko Zaw, Si Thu Aung

Résumé
Contexte: Des organisations internationales non gouvernementales (OING) mettent en œuvre des initiatives communautaires de traitements de la tuberculose (ICTTB) au Myanmar depuis 2011. Bien que le Programme national antituberculeux (PNA) est censé de complètement prendre la relève des ICTTB, il n'y a eu aucune évaluation des modèles de soins ni des coûts relatifs aux ICTTB réalisées par des OING au Myanmar.
Méthodes: Il s'agit d'une étude descriptive à l'aide de données financières et programmatiques cueillies systématiquement de quatre OING en 2013 et 2014, corrigées en fonction de l'inflation. Il y a eu une analyse des données du point de vue du fournisseur. Les coûts liés aux analyses des expectorations ne sont pas compris, puisqu'elles étaient réalisées gratuitement dans le cadre du PNA. Nous avons calculé le coût moyen par année pour chaque programme et le coût par patient qui suit le traitement.
Résultats: Quatre OING ont aidé dans le cadre du PNA en réalisant des ICTTB dans des régions où l'accès aux traitements de la tuberculose était difficile. Chaque OING a dû faire face à des enjeux différents, selon le contexte, et a surmonté les difficultés à l'aide de diverses stratégies. Le coût total se situait entre 140 754 $ US et 550 221 $ US pendant la durée de l'étude. Le coût par patient qui a suivi le traitement se situait entre 215 $ US et 1 076 $ US pour les nouveaux cas, et entre 354 $ US et 1 215 $ US pour les reprises de traitement, selon la région ciblée et l'ensemble des services offerts. Un OING semblait moins cher, plus durable et plus axé sur les patients que les autres.
Conclusions: Les résultats de l'étude ont fait ressortir un vaste éventail de modèles de soins et de coûts connexes à la mise en œuvre d'ICTTB dans des populations et des contextes divers et présentant des défis au Myanmar. Par conséquent, nous conseillons qu'une évaluation plus exhaustive soit réalisée, y compris l'élaboration d'un modèle des coûts, pour arriver à une estimation des coûts relatifs au déploiement d'ICTTB à l'échelle du pays, et une étude coût-efficacité pour mieux éclairer les PNA, alors qu'ils s'apprêtent à prendre la relève des ICTTB des OING. En attendant les résultats de ces études, le soutien prioritaire devrait être accordé au modèle d'ICTTB qui présente le meilleur potentiel de durabilité, et plus de ressources devraient être allouées aux soins axés sur les patients.

Translated from English version into French by Edith Emilie Mercier, through

TRANSLATORS WITHOUT BORDERS

Задачи, решения и связанные с этим расходы при лечении территориального туберкулеза на базе международных негосударственных организаций в Мьянме

Вай Вай Хан, Соу Соу, Петрос Исаакидис, Мохаммед Кхогали, Энтони Рейд, Нгуен Хоа, Ко Ко Зоу, Си Ту Аунг

Краткий обзор
Основание: Международные негосударственные организации (МНГО) реализуют лечение территориального туберкулеза (ТБ) в Мьянме с 2011 года. Хотя, в конечном счёте, национальная программа лечения туберкулёза (NTP) планирует взять на себя роль МНГО, оценок моделей ухода или стоимости лечения туберкулёза в Мьянме не проводилось.
Методы: Описательное исследование с использованием регулярно собираемых программных и финансовых данных из четырех МНГО в 2013 и 2014 годах, с поправкой на инфляцию. Анализ данных проводился с точки зрения поставщика услуг. Расходы на анализы мокроты не были включены, поскольку предоставляется бесплатно НГО. Мы подсчитали среднюю стоимость в год каждой программы и расходы на завершение лечения пациента.
Результаты: Четыре МНГО оказали содействие NTP путем предоставления услуг в лечении туберкулёза в районах, где затруднён доступ к аналогичным услугам. Все МНГО столкнулись с различными проблемами и решали их разнообразными методами. Общие расходы варьировались от 140754 до 550221 долларов США в течение периода исследования. Стоимость завершения лечения пациента варьировалась от 215 до 1076 долларов США для новых случаев и от 354 до 1215 долларов США для возобновление лечения, в зависимости от региона и предоставляемого пакета услуг. Одно

МНГО проявило себя более экономным, самодостаточным и ориентированным на пациентов, чем другие.
Заключение: Данное исследование показало широкий спектр моделей лечения и связанных с этим расходов по реализации лечения туберкулёза в многообразных группах населения в Мьянме. Таким образом, рекомендуется провести всеобъемлющую оценку, включая разработку модели стоимости, для оценки расходов по расширению предоставления услуг по лечению туберкулёза в масштабах всей страны, а также изучение экономической эффективности для лучшего информирования NTP в рамках перехода контроля по лечению туберкулёза к NTP. В ожидании результатов данных исследований, следует оказать приоритетную поддержку модели лечения туберкулёза, имеющей наиболее высокий потенциал самодостаточности и ориентированной на пациентов.

Translated from English version into Russian by Semen Abramov, through

Diferentes desafíos, diferentes enfoques y gastos conexos de las actividades comunitarias de lucha contra la tuberculosis por parte de organizaciones internacionales no gubernamentales en Myanmar

Wai Wai Han, Saw Saw, Petros Isaakidis, Mohammed Khogali, Anthony Reid, Nguyen Hoa, Ko Ko Zaw, Si Thu Aung

Resumen
Antecedentes: Las organizaciones internacionales no gubernamentales (ONGIs) han venido prestando atención comunitaria (CBTBC) contra la tuberculosis (TB) en Myanmar desde el año 2011. Aunque el Programa Nacional de Tuberculosis (NTP) planea tomar el control de la CBTBC, no se han realizado evaluaciones de los modelos de atención o de los costos de proporcionar CBTBC en Myanmar por parte de las ONGs.
Métodos: Se trata de un estudio descriptivo utilizando datos programáticos y financieros recopilados rutinariamente, de cuatro ONGIs durante 2013 y 2014, y ajustados por inflación. El análisis de los datos se realizó desde la perspectiva del proveedor. No se incluyeron los costos para el examen de esputo ya que fue proporcionado gratuitamente por el NTP. Se calculó el costo promedio por año de cada programa y el costo por paciente que completaba el tratamiento.
Resultados: Cuatro ONGI ayudaron al NTP proporcionando CBTBC en áreas donde el acceso a los servicios para la TB era un desafío. Cada ONGI hizo frente a diferentes problemas en sus contextos y respondió con una diversidad de estrategias. Los costos totales oscilaron entre US$ 140754 y US$ 550221 durante el período de estudio. El costo por paciente que completó el tratamiento varió de US$ 215 a US$ 1076 para casos nuevos y de US$ 354 a US$ 1215 para casos de retratamiento, dependiendo del área y el paquete de servicios ofrecidos. Una ONGI aparece menos costosa, más sostenible y orientada al paciente que otras.
Conclusiones: Este estudio reveló una amplia variedad de modelos de atención y costos asociados para implementar la CBTBC en poblaciones y contextos diversos y desafiantes en Myanmar. Por consiguiente, recomendamos una evaluación más exhaustiva, que incluya el desarrollo de un modelo de costos, para estimar los costos de ampliar la CBTBC a todo el país y estudios de costo-efectividad, para informar mejor al NTP ya que se prepara para asumir las actividades de CBTBC de las ONGIs. Mientras se espera evidencia de estos estudios, se debe dar prioridad al modelo de CBTBC que tiene mayor potencial de sostenibilidad y asignar más recursos a la atención centrada en el paciente.

Translated from English version into Spanish by patriciacassoni, through
